# FGFR3b Extracellular Loop Mutation Lacks Tumorigenicity *In Vivo* but Collaborates with p53/pRB Deficiency to Induce High-grade Papillary Urothelial Carcinoma

**DOI:** 10.1038/srep25596

**Published:** 2016-05-09

**Authors:** Haiping Zhou, Feng He, Cathy L. Mendelsohn, Moon-shong Tang, Chuanshu Huang, Xue-Ru Wu

**Affiliations:** 1 Departments of Urology, Pathology and Enviromental Medicine, New York University School of Medicine, New York, New York 10016, USA.; 2 Departments of Urology, Genetics & Development, Columbia University, New York, New York 10032, USA.; 3 Veterans Affairs New York Harbor Healthcare System, Manhattan Campus, New York, New York 10010, USA

## Abstract

Missense mutations of fibroblast growth factor receptor 3 (FGFR3) occur in up to 80% of low-grade papillary urothelial carcinoma of the bladder (LGP-UCB) suggesting that these mutations are tumor drivers, although direct experimental evidence is lacking. Here we show that forced expression of FGFR3b-S249C, the most prevalent FGFR3 mutation in human LGP-UCB, in cultured urothelial cells resulted in slightly reduced surface translocation than wild-type FGFR3b, but nearly twice as much proliferation. When we expressed a mouse equivalent of this mutant (FGFR3b-S243C) in urothelia of adult transgenic mice in a tissue-specific and inducible manner, we observed significant activation of AKT and MAPK pathways. This was, however, not accompanied by urothelial proliferation or tumorigenesis over 12 months, due to compensatory tumor barriers in p16-pRB and p19-p53-p21 axes. Indeed, expressing FGFR3b-S249C in cultured human urothelial cells expressing SV40T, which functionally inactivates pRB/p53, markedly accelerated proliferation and cell-cycle progression. Furthermore, expressing FGFR3b-S243C in transgenic mouse urothelium expressing SV40T converted carcinoma-*in-situ* to high-grade papillary urothelial carcinoma. Together, our study provides new experimental evidence indicating that the FGFR3 mutations have very limited urothelial tumorigenicity and that these mutations must collaborate with other genetic events to drive urothelial tumorigenesis.

Urothelial carcinoma of the bladder (UCB) ranks the fourth among the most common cancers in American men with the lifetime risk of 1 in 26^1^. Of all the UCB, 70–75% present as low-grade, papillary urothelial carcinoma of the bladder (LGP-UCB; or stage Ta) that are frequently multifocal and recurrent after initial surgical removal and concurrent intravesicle chemotherapy^2^,^3^,^4^,^5^. Additionally, 20–25% of the LGP-UCB are believed to progress over time in grade and/or stage to high-grade, invasive UCB^6^,^7^,^8^,^9^. Such risks of recurrence and progression often necessitate vigilant and long-term follow-up of LGP-UCB with repeated diagnostic procedures and increasingly aggressive therapeutic modalities. This is one of the main reasons why UCB is the costliest cancer to manage clinically on a per-case basis^10^,^11^.

Among the genetic alterations identified to date in LGP-UCB, mutations involving the fibroblast growth factor receptor 3 (FGFR3) gene are by far the most common, occurring in up to 80% of all the tumors^12^,^13^. The great majority of these mutations belong to missense point mutations that alter a single amino acid residue. Although the mutations can reside in the extracellular loops, the transmembrane domain or the cytoplasmic kinase domains of FGFR3, those in the extracellular loops are the most common^14^,^15^. Interestingly, the cysteine-altering mutations, i.e., mutations that either abrogate an original cysteine or create a new one, dominate the extracellular loop mutations. It has been suggested that these mutations can lead to the mispairing of the cysteines between two FGFR3 protein molecules and, consequently, ligand-independent receptor dimerization and activation^16^. Whether these cysteine-altering FGFR mutants become misfolded and trapped in the endoplasmic reticulum entirely and activate downstream signals intracellularly, or some of them can still translocate to the cell surface and be partially ligand-dependent during urothelial transformation and tumorigenesis remains unresolved^17^. On the other hand, non-cysteine mutations in the transmembrane and cytoplasmic domains of FGFR3 are believed to change the conformational structure of the kinase domains, leading to their constitutive activation. Nevertheless, these mutations can remain somewhat ligand-dependent.

The sheer abundance of the FGFR3 mutations in LGP-UCB implies that they may play an important role in urothelial tumorigenesis^18^, although direct experimental evidence remains scarce. On the one hand, enforced expression of various FGFR3 mutants in cultured urothelial cells could activate MAPK pathway and promote cell proliferation and survival^16^,^19^, effects that are known to be conducive to tumorigenesis. On the other hand, expression of a kinase mutant (K652E) of FGFR3 in the urothelium of transgenic mice failed to elicit urothelial proliferation, let alone tumor formation after 18 months, thus refuting a tumorigenic role of this particular mutation^20^. The tumorigenicity or, the lack thereof, of the more prevalent, cysteine-altering extracellular loop mutations in an *in vivo* physiologically relevant system has not been addressed.

In the present study, we examined the tumorigenic potential of a cysteine-altering mutant of FGFR3 (e.g., S249C), the most prevalent FGFR3 mutation in human LGP-UCB^14^,^15^, using a combination of cultured cells and transgenic mice. We assessed the pro- as well as the anti-proliferative responses of the urothelial cells to this FGFR3 mutant. We further tested the effects of suppressing the anti-proliferative responses elicited by the mutated FGFR3 on urothelial tumor formation. Our results provide direct *in vivo* evidence indicating that FGFR3 mutations by themselves have very limited tumorigenic activity and they require specific collaborative events, particularly deficiencies in one or more tumor suppressive pathways, in order to initiate urothelial tumors. Based on the data presented here and those published previously, we provide an integral view of the role of RTK/RAS pathway components in the genesis and progression of major phenotypic variants of urothelial carcinoma of the bladder.

## Results

### The Extracellular Loop Mutant of FGFR3b-S249C Accelerated Urothelial Proliferation *in vitro*

**Figure 1:**
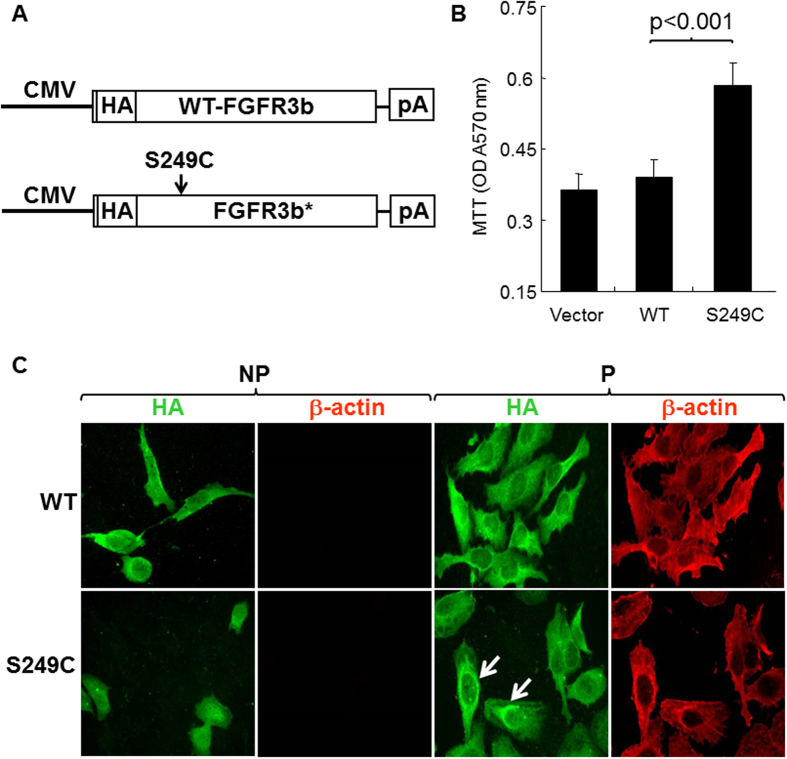
Expression and activity of FGFR3b-S249C mutant in cultured urothelial cells. (**A**) Wild-type (WT) FGFR3b cDNA and mutant FGFR3b-S249C cDNA that were constructed to contain the hemagglutinin (HA) tag were separately cloned downstream of a CMV promoter in pcDNA3.1 mammalian expression vector. pA represents poly A signal. (**B,C**) The vector alone or those containing the cDNAs were transfected into the UMUC3 cell line, which expressed little endogenous FGFR1, 2, 3 or 4 (data not shown). Stably transfected cells, after being re-plated for 48 hours, were subjected to cell proliferation (MTT) assay (**B**) or immunofluorescent double staining using anti-HA and anti-β-actin (as an indicator for cytoplasmic staining) under non-permeabilized (NP) or permeabilized condition (P) (panel (**C**). Note that the expression of the FGFR3b-S249C mutant, but not the vector only or the vector containing the wild-type FGFR3b, significantly increased the cell proliferation. Also note that, while the FGFR3b-S249C mutant exhibited significant perinuclear staining in permeabilized cells, some of it could still be detected on the cell surface of non-permeabilized cells. Magnification in (**C**), 200x.

To test the activity of our cDNA construct containing the FGFR3b-S249C mutant and to set a stage for *in vivo* transgenic mouse work, we first performed *in vitro* expression experiments using cultured urothelial cells. We devised an isogenic system comprising a human FGFR3b-S249C cDNA and a human urothelial host cell line (e.g., UMUC3), which expressed little endogenous FGFR1, 2, 3 and 4 (data not shown). The detection of the FGFR3-S249C mutant was facilitated by the insertion of an *Influenza* virus hemagglutinin (HA) tag between amino acid residues 27 and 28, a region preceding the extracellular loop domains of FGFR3b (Fig. 1A) where there was little predicted secondary structure. Wild-type FGFR3b cDNA and empty vector were used as controls. While the enforced expression of the wild-type FGFR3b cDNA only slightly increased the proliferation of UMUC3 cells over the vector control, that of the FGFR3b-S249C mutant increased the proliferation by nearly 2-fold (p < 0.001; Fig. 1B). Immunofluorescent staining using anti-HA of transfected, non-permeabilized UMUC3 cells (that were therefore negative for intracellular β−actin staining) showed cell surface labeling for the wild-type FGFR3b and less so for the FGFR3b-S249C mutant (Fig. 1C). Immunofluorescent staining using anti-HA of transfected, permeabilized UMUC cells showed perinuclear aggregates of the FGFR3b-S249C mutant (Fig. 1C; arrows). This is consistent with the notion that at least some of the FGFR3b-S249C mutants could dimerize precociously via the *de novo* cysteine, thus being misfolded and trapped in the endoplasmic reticulum.

### Tissue-specific and Inducible Expression of FGFR3b Mutant in Transgenic Mouse Urothelium

**Figure 2:**
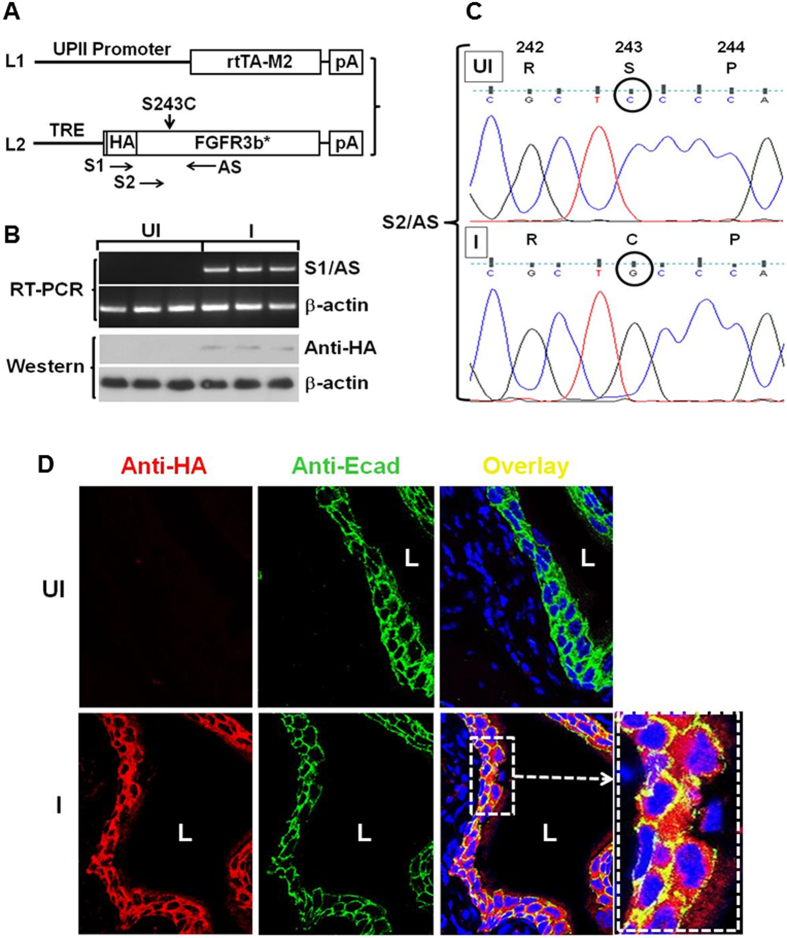
Urothelium-specific and inducible expression of FGFR3b-S243C mutant in transgenic mice. (**A**) Transgene constructs. Line 1 (L1) represented a previously generated transgenic mouse line in which the 3.6-kB mouse uroplakin II (UPII) promoter drove the expression of a modified reverse tetracycline transactivator (rtTA-M2). pA, poly A signal. Line 2 (L2) was a newly generated transgenic mouse line in which the tetracycline responsive elements (TRE) drove the expression of hemagglutinin (HA)-tagged mouse FGFR3b-S243C mutant cDNA. S1, S2 and AS were oligonucleotide primers used for RT-PCR and sequencing (see panels (**B,C**)). (**B**) RT-PCR and Western blotting of total RNAs and total proteins, respectively, from the double transgenic mice harboring both UPII-rtTA-M2 and TRE-FGFR3b-S243C transgenes. UI, un-induced mice (i.e., mice placed on a regular diet for 14 days); I, induced mice (i.e., mice placed on doxycycline-containing diet for 14 days). Three representative mice were shown for each condition. Note the specific detection of the FGFR3b-S243C on RNA and protein levels only in the induced mice. (**C**) Direct sequencing of the RT-PCR products from the S2/AS primer amplification showed the un-mutated codon 243 (TCC encoding serine (S)) in un-induced double transgenic mice, representing the endogenous wild-type FGFR3b; and the mutated codon 243 (TGC encoding cysteine (**C**)) in induced mice, representing the transgene product. Only three codons were shown and the rest of the regions were all identical. (**D**) Double immunofluorescent staining using anti-HA and anti-E-cadherin (Ecad) showing the lack of expression of the FGFRb-S243C in un-induced mice or the strong expression of the mutant in induced mice (induction for 14 days). Also note the presence of both cytoplasmic and surface membrane staining of FGFR3b-S243C (overlapped with anti-Ecad; highlighted in dotted boxes). Magnification in (**D**), 200x.

To determine the *in vivo* tumorigenic potential of the FGFR3b mutant, we engineered the FGFR3b mutant cDNA downstream of a tetracycline responsive element (TRE-FGFR3b*), and then crossed the transgenic mice with another transgenic line that we generated previously that expressed a modified tetracycline transactivator under the control of a mouse uroplakin II promoter (UPII-rtTA-M2) (Fig. 2A 21;). We again devised an isogenic system, i.e., expression of a mouse version of HA-tagged FGFR3b mutant (S243C) in mice (Fig. 2A), to ensure ligand-receptor interaction if the mutant can partially translocate onto the cell surface.

The double transgenic mice harboring both the UPII-rtTA-M2 and the TRE-FGFR3b-S243C transgenes were allowed to reach adulthood (e.g., 2 months of age) and randomized into two groups, one receiving regular food pellets and another receiving food pellets containing doxycycline (20 g/kg). RT-PCR and Western blotting showed the expression of FGFR3b-S243C mutant in doxycycline-treated mice, but not in non-treated controls (Fig. 2B). Direct sequencing of the RT-PCR products confirmed the presence of the mutation on codon 243 converting the wild-type serine to cysteine, only in doxycycline-treated mice (Fig. 2C). FGFR3b-S243C expression was not detected by RT-PCR in any non-urothelial tissues of double transgenic mice treated with doxycycline (data not shown). Immunofluorescent staining using anti-HA antibody showed the complete absence of expression of the FGFR3b-S243C in un-induced double transgenic mice and the strong expression in induced double transgenic mice (Fig. 2D). Interestingly, while intracellular labeling of FGFR3b-S243C was apparent as expected, cell surface labeling was also noticeable as evidenced by co-localization using an anti-E-cadherin antibody. These results established the successful generation of a transgenic mouse system that allowed urothelium-specific and time-controlled expression of the FGFR3b-S243C mutant.

### Lack of Urothelial Tumorigenicity of the Extracellular Loop Mutant of FGFR3b-S243C Was Due Partly to Compensatory Tumor Barriers

**Figure 3:**
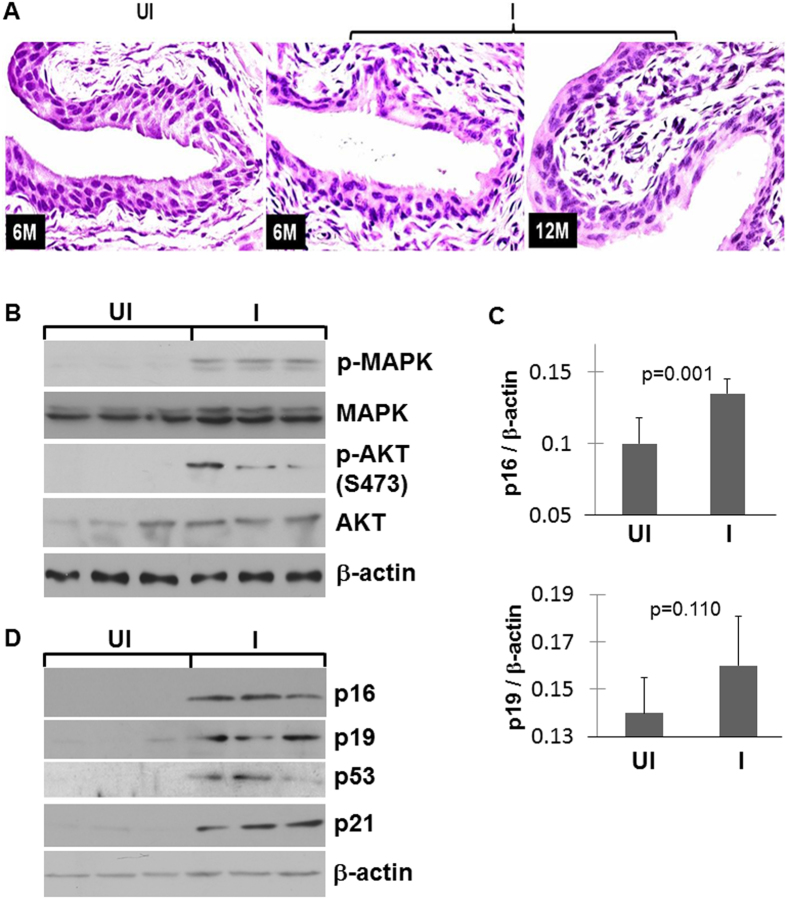
Morphological and molecular characterization of transgenic mice expressing FGFR3b-S243C. (**A**) Representative H&E-stained images of urothelial cross-sections from un-induced double transgenic mice (UI, 6 months old) and induced (I) double transgenic mice (6 and 12 months old). Note the complete absence of urothelial abnormality in the induced mice. Magnification, 200x. (**B**) Western blotting showing the upregulation of phosphorylated MAPK and AKT in induced mice, as compared with uninduced mice (3 mice each condition). (**C**,**D**) Real-time PCR and Western blotting showing the upregulation of p16-pRb and p19-p53-p21 tumor suppressor pathway components. N = 5 in (**C**).

We next followed a cohort (n = 60) of doxycycline-treated double transgenic mice urothelially expressing the FGFR3b-S243C for 12 months by sacrificing 5 mice every two months and examining the histopathology of the urinary bladders. To our surprise, urothelia of all the mice, even at the last time point (12 months) appeared completely normal and indistinguishable from the double transgenic mice without doxycycline treatment (Fig. 3A). This occurred despite the fact that both MAPK and AKT pathways were significantly activated in the induced (doxycycline-treated) mice, as evidenced by the increased levels of phosphorylated MAPK and AKT (Ser473; Fig. 3B). We surmised that the compensatory induction of tumor barriers via an overexpression of tumor suppressor genes may contribute to the lack of tumorigenicity by the FGFR3 mutation, and therefore surveyed a number of such targets using real-time quantitative PCR. We found genes operative in the p16-pRb and p19-p53-p21 pathways to be significantly upregulated on mRNA and protein levels in urothelial cells expressing the FGFR3b-S243C mutant, but not in non-expressing controls (Fig. 3C,D). These results raised the interesting possibility that the oncogenic effects of the FGFR3b mutant in urothelial cells may be negated by the secondary tumor defenses, thus rendering the FGFR3b non-tumorigenic.

### Disabling p16-pRb and p19-p53-p21 Pathways Accelerated Urothelial Proliferation *in vitro* and Induced Urothelial Tumorigenesis *in vivo*

**Figure 4:**
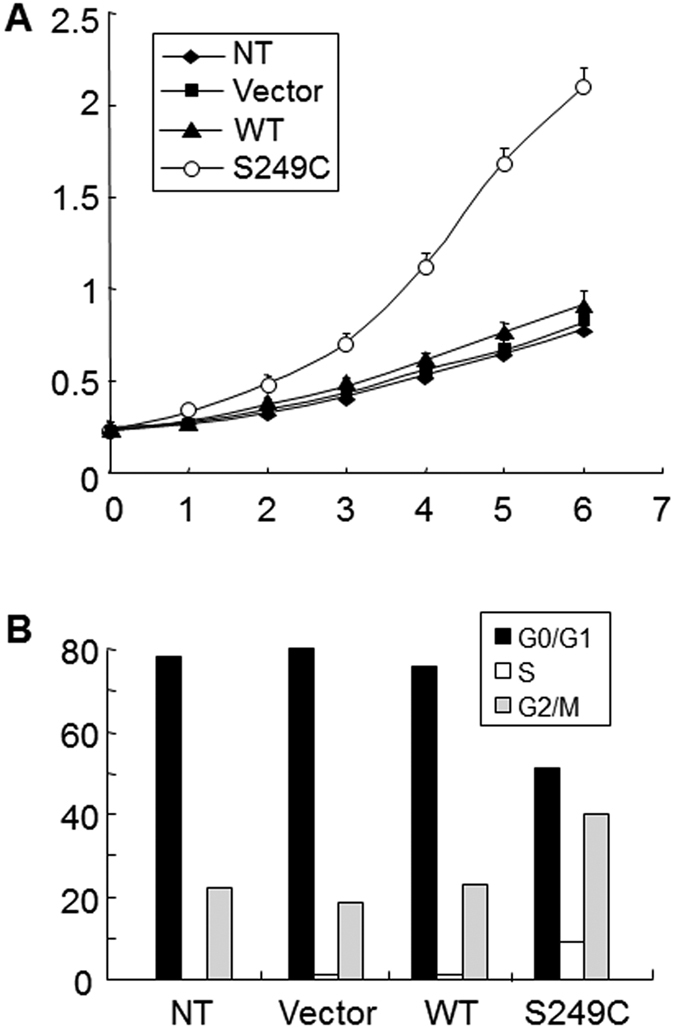
Acceleration of proliferation and cell cycle progression by FGFR3b-S249C in urothelial cells expressing SV40 large T antigen. Mammalian expression vector or that containing the wild-type (WT) FGFR3b or that FGFR3b-S249C mutant was transfected into UROtsa, a human urothelial cell line constitutively expressing SV40 large T antigen. Non-transfected (NT) cells were used as a control. Stably transfected cells were regrown for 12, 24, 36, 48, 60 and 72 hours (represented by 1–6 time points, respectively) and harvested for cell proliferation (MTT) assay (**A**). Note that the cells transfected with FGFR3b-S249C showed significantly increased proliferation. Additionally, the cells grown for 48 hours were subject to cell cycle analysis with fluorescence-activated cell sorting after staining by propidium iodide (**B**). Note that the cells transfected with FGFR3b-S249C had significantly decreased presence in G0/G1 and significantly increased presence in S and G2/M phases.

If the upregulation of the molecular players in the p16-pRb and p19-p53-p21 pathways indeed acted as tumor barriers in urothelial cells expressing the FGFR3 mutant, their removal should then enhance urothelial proliferation or even induce urothelial tumor formation. To test this hypothesis, we first transfected a mammalian expression vector containing the human FGFR3b-S249C mutant cDNA into human urothelial cells that were immortalized with the SV40T antigen (the latter of which is known to bind and functionally inactivate both p53 and pRb^22^;). While the expression of the wild-type FGFR3b did not promote cell proliferation beyond the non-transfected or vector-transfected controls in UROtsa cells, the FGFR3-S249C mutant markedly increased cell proliferation (Fig. 4A). This corresponded with a significant increase of the mutant-transfected cells in the S and G2/M phases (Fig. 4B).

**Figure 5:**
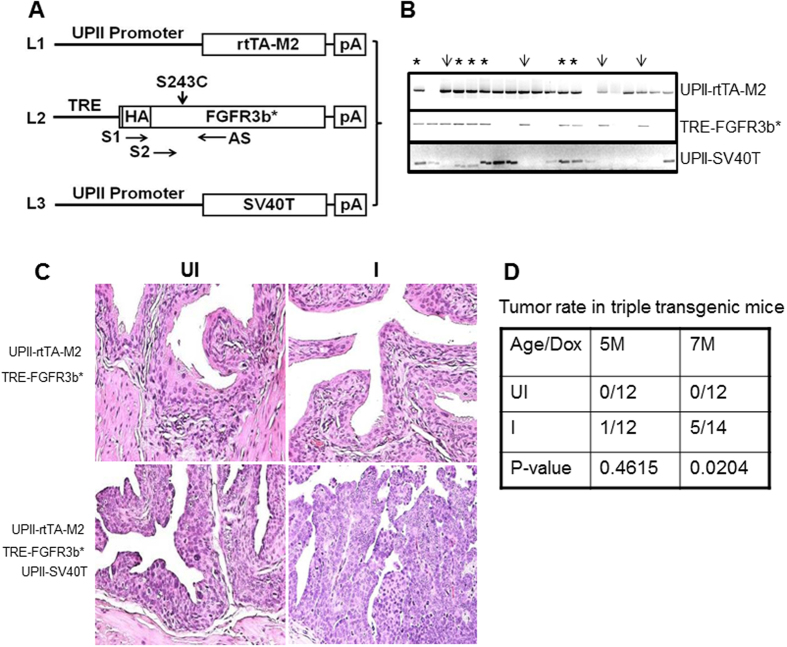
Formation of high-grade, papillary urothelial carcinoma of the bladder in double transgenic mice expressing both FGFR3b-S243C and SV40T. (**A**) Schematic diagram of compound transgenic lines. Lines 1 (L1) and 2 (L2) were those depicted in Fig. 2 and line 3 (L3) was a previously generated transgenic line harboring low-copy-numbered UPII-SV40T transgene and by itself exhibiting consistently carcinoma-*in-situ* lesions between 1–10 months of age. (**B**) Intercrossing the three lines shown in (**A**) and further intercrossing of the siblings yielded double transgenic mice bearing the UPII-rtTA-M2 and the TRE-FGFR3b-S243C transgenes (thin arrows), and triple transgenic mice bearing the two aforementioned transgenes, plus the UPII-SV40T transgene (asterisks). These two groups of compound mice were each randomized into two groups, one receiving a regular diet (**C**,**D**; UI) and another doxycycline-containing diet (**C,D**; I). At 5 and 7 months post-treatment, all mice were sacrificed and their bladders procured for histopathological examination. (**C**) Representative images of H&E-stained bladders of the two groups of compound mice with or without doxycycline induction. Note the normal-appearing urothelium in the double transgenic mice with or without doxycycline. Also note the high-grade carcinoma-*in-situ* lesion in the triple transgenic mice without doxycycline induction. and the high-grade, papillary bladder tumor in the triple transgenic mice with doxycycline induction. Magnification, 200x. (**D**) A summary of tumor frequency in triple transgenic mice.

**Figure 6:**
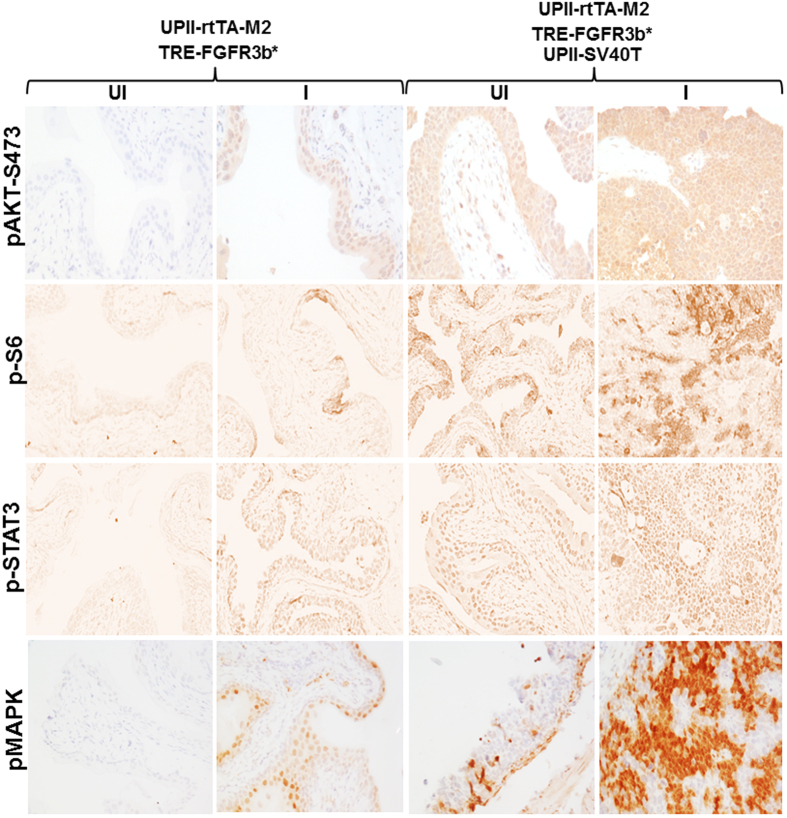
Signaling pathway status in triple transgenic mice expressing both FGFR3b-S243C and SV40T. Paraffin-embedded sections from the bladders of transgenic mouse groups shown in Fig. 5 were processed routinely for immunohistochemical staining using the antibodies indicated on the left. Note the marked upregulation of both MAPK and AKT pathways, particularly phosphorylated S6 and phosphorylated MAPK in triple transgenic mice treated with doxycycline (the right most panel). Magnification, 200x.

To extend these findings *in vivo*, we cross-bred the double transgenic mice bearing the UPII-rtTA-M2 and the TRE-FGFR3b-S243C transgenes with the low-copied, UPII-SV40T transgenic mice that we generated earlier^23^,^24^. After additional crosses among the siblings, the resultant double (UPII-rtTA-M2 and TRE-FGFR3b-S243C) and triple (UPII-rtTA-M2, TRE-FGFR3b-S243C and UPII-SV40T) transgenic mice (Fig. 5A,B) at 2 months of age either continued to receive the regular diet or were placed on doxycycline-containing diet continuously to induce FGFR3b-S243C expression. The above four groups of mice were sacrificed at 5 and 7 months post-treatment and their urinary bladders subjected to histopathological examination. Consistent with our initial study of the UPII-rtTA-M2 and TRE-FGFR3b-S243C double transgenic mice (Fig. 3), the urothelia appeared normal with or without the induction of FGFR3b-S243C expression (Fig. 5C). Without doxycycline treatment (e.g., without FGFR3b-S243C expression), the triple transgenic mice bearing UPII-rtTA-M2, TRE-FGFR3b-S243C and UPII-SV40T transgenes exhibited high-grade carcinoma-*in-situ* lesions in the bladder (Fig. 5C), much like what was observed reproducibly in low-copied, UPII-SV40T single transgenic mice^23^,^24^. In contrast, doxycycline-treated triple transgenic mice (with FGFR3b-S243C expression) harbored high-grade papillary bladder tumors resembling human pTaG2–3 tumors (Fig. 5C,D). It seems clear therefore that the functional inactivation of pRb and p53 by SV40T could collaborate with FGFR3b mutation to trigger urothelial tumors *in vivo*. Immunohistochemical analyses further showed that the increased levels of phosphorylated MAPK and AKT, but not that of STAT3, was associated with the formation of the high-grade, papillary bladder tumors in the triple transgenic mice (Fig. 6). This suggested that the inactivation of pRb and p53 allowed the downstream signals of FGFR3b to overactivate, helping fuel urothelial proliferation and tumor formation.

## Discussion

### Mutational Activation of FGFR3b Alone Is Non-tumorigenic in Urothelium

A key finding of our present study was that the urothelium-specific and time-controlled expression of an FGFR3b mutant (S243C) alone in transgenic mice was insufficient to trigger urothelial tumorigenesis (Fig. 3). This was largely unexpected because the mutant we chose is by far the most prevalent among all the FGFR3b mutations in human urothelial carcinoma of the bladder (UCB)^14^,^15^; because the mutant is considered ligand-independent and constitutively active^16^,^17^; and because the mutant exerted significant pro-proliferative effects in cultured urothelial cells as shown here (Fig. 1) and by other investigators previously^16^,^19^. Our results, coupled with those showing that a kinase mutant of FGFR3b (K652E) alone also failed to cause urothelial tumor formation in transgenic mice^20^, strongly suggests that the mutational activation of FGFR3b by itself lacks significant tumorigenicity in urothelial cells *in vivo*. These two independent studies, both based on biologically relevant systems, therefore do not support the conventional belief that, since FGFR3b mutations are extremely prevalent in human UCB, they are the molecular drivers for these tumors^25^.

It should be emphasized that the lack of tumorigenicity of the FGFR3b mutant *in vivo* was not due to the lack of growth-promoting/oncogenic activities of the mutant *per se*. As we found out, the FGFR3b S249C mutant actually significantly activated both MAPK and AKT (Figs 1 and 3), signals critical for tumorigenesis in urothelial as well as non-urothelial cells^26^. It is, however, the compensatory tumor defense of the urothelial cells that counteracted the growth-promoting effects of the FGFR3 mutant, rendering the mutant non-tumorigenic. In particular, key components of the p16-pRB and p19-p53-p21 tumor suppressor pathways, that were barely detectable in normal urothelial cells, were strongly upregulated in FGFR3b mutant-expressing cells (Fig. 3). Such compensatory tumor defenses have been observed with other oncogenic events, such as RAS activation^27^ or deficiency of pRb^28^ and PTEN^29^, when they are targeted into the urothelial cells *in vivo*. These urothelial tumor defenses therefore appear to be more of a rule than an exception, and should be kept in mind when extrapolating the *in vivo* effects, or the lack therefore, of genetic and molecular alterations on urothelial tumorigenesis (see below).

### Combinatorial Genetic Drivers of High-grade Papillary Urothelial Carcinoma

In direct support of our tumor-defense line of reasoning, functional inactivation of both p16-pRB and p19-p53-p21 pathways by urothelial expression of SV40 large T antigen induced high-grade papillary urothelial carcinoma of the bladder (HGP-UCB) in double transgenic mice that simultaneously expressed the FGFR3b mutant in the urothelium (Figs 3, 4, 5, 6). Thus, FGFR3b mutation and pRb/p53 deficiency apparently act synergistically as combinatorial genetic drivers of HGP-UCB in our experimental system. Since single transgenic mice expressing SV40T-only consistently developed carcinoma *in situ*^23^,^30^, the formation of HGP-UCB in the double transgenic mice could be a result of the acquisition of increased growth potential conferred by the FGFR3 mutant, which was missing in the SV40T single transgenic mice. Interestingly, similar collaborative relationships existed between SV40T and overexpressed EGFR^31^, and between SV40T and activated HRAS^26^. Each of these genetic combinations led to the formation of HGP-UCB.

Although, in humans, HGP-UCB (or pTaG3) accounts for a relatively small proportion (2.9 to 18.0%) of all UCB, it is a highly important clinical entity due to its propensity to progress to the invasive stage^8^,^9^,^32^,^33^. The molecular drivers underlying human HGP-UCB remain poorly characterized to date. A limited number of studies found that HGP-UCB harbor significantly more chromosomal abnormalities (e.g., gains and deletions) than the low-grade counterpart^34^, suggesting that HGP-UCB are more genomically unstable and prone to progress. Results from our present study therefore offer potential leads to further studying whether a combination of activation of receptor tyrosine kinase/RAS pathway and inactivation of pRb/p53 also plays an important role in the genesis of HGP-UCB in humans.

### Role of Genetically Engineered Mouse Models in Defining Tumor Drivers

With vastly improved instrumentation and reduced cost it is now possible to profile the genetic and molecular characteristics of large cohorts of human tumors. The field of urothelial carcinoma has recently made significant inroads in this arena as well. For instance, whole-exome sequencing and multiplatform analyses have revealed new molecular signatures of not only the major types of urothelial carcinomas, but also clinically meaningful subtypes that were not recognized before^35^,^36^,^37^,^38^,^39^,^40^,^41^,^42^. Notwithstanding these important advances, it should be pointed out that the molecular phenotypes of a given tumor type or subtype identified in human specimens does not necessarily equate their being the tumor drivers. In fact, many key components of a molecular profile, such as cytokeratins, uroplakins and cadherins^40^,^41^,^42^, are actually the consequence, rather than the cause, of tumor formation. The fact that even a genetic alteration as prevalent as FGFR3 mutation is by itself incapable of causing urothelial carcinoma *in vivo*, as we demonstrated here, illustrates the need to validate the molecular profiling data from human tumors using experimental systems. In this regard, the genetically engineered mouse models (GEMMs) will continue to play a pivotal role in better defining the biological effects of the molecular alterations identified in human patients. GEMMs can be highly informative in discerning the tumor drivers from the passengers; establishing or refuting the collaborative relationships among divergent molecular alterations; and separating the genetic alterations responsible for tumor initiation versus those for tumor progression^43^. Human-relevant GEMMs should continue to provide important toolboxes for evaluating novel ideas for treating and preventing urothelial carcinoma of the bladder^19^,^44^,^45^,^46^. The complementary and bidirectional flow of information from human to mouse and back to human again should continue to be the paradigm to eventually elucidating the molecular bases underlying the divergent forms of urothelial carcinoma and their interrelationships and modes of progression.

## Materials and Methods

### Expression Vectors, Cell Lines and Stable Transfection

A full-length cDNA encoding human wild-type FGFR3b (Addgene) was cloned into mammalian expression vector pcDNA3.1 and used as a template for site-directed mutagenesis to (i) add a hemagglutinin (HA) tag to facilitate specific detection and (ii) create the FGFR3b-S249C mutation using QuikChange site-directed mutagenesis kit (Stratagene). The mutagenesis primers for HA addition were: forward: 5′-CCTCGGAGTCCTTGGGGACGTACCCATACGATGTTCCAGATTACGCTGAGCAGCGCGTCGTGGGGCG-3′; and reverse: 5′-CGCCCCACGACGCGCTGCTCAGCGTAATCTGGAACATCGTATGGGTACGTCCCCAAGGACTCCGAGG-3′. Those for FGFR3b-S249C mutagenesis were: forward: 5′-CGTGCTGGAGCGCTGCCCGCACCGGCCC-3′; and reverse: 5′-GGGCCGGTGCGGGCAGCGCTCCAGCACG-3′. The cDNA insert was fully sequenced to confirm the authenticity and the presence of the newly added HA and the mutation site. UMUC3, a human urothelial carcinoma cell line, was purchased from ATCC and cultivated in the presence of Eagle’s Minimum Essential Medium and 10% fetal bovine serum. UROtsa, an SV40 large T antigen-immortalized human urothelial cell line (courtesy of Dr. Ricardo Saban of University of Oklahoma) was maintained in Dulbeco’s modified Eagle’s medium and Ham’s F-12 supplemented with 10% fetal bovine serum.

For transfection experiments, 5 × 10^4^ cells/well were seeded into 24-well cell culture plates. After 16 h, the attached cells were transfected using Lipofectamine 2000 (Invitrogen) with the vector only, that containing wild-type FGFR3b or that containing FGFR3b-S249C. After 48 h, a selection medium containing G418 (1 mg/ml) was added. The surviving cells continued to be cultured in this medium for 4 additional weeks, after which single clones were selected and expanded into stable lines.

### Proliferation and Cell Cycle Analysis and Immunofluorescent Staining of Cultured Cells

For cell proliferation analysis, 5 × 10^3^/well stably transfected cells were seeded in 96-well cell culture plates. After 48 h of incubation, MTT (3-(4,5-dimethylthiazol-2-yl)-2,5-diphenyltetrazolium bromide; 5 mg/ml in PBS) was added to each well. After incubation at 37 °C for 2 h, 0.1 ml lysis buffer (20% SDS and 50% dimethylformamide) was added, and the lysates were subject to spectrophotometry at 570 nm.

For cell cycle analysis, non-transfected and stably transfected cells were first cultured in regular media (see above) for 24 h and then switched to serum-free media and cultured for 6 h. Cells were harvested, fixed in 100% ice-cold ethanol, washed and treated with 1 mg/ml propidium iodide with RNase A (200 μg/ml). Cell cycle distribution was analyzed by fluorescence-activated cell sorting (FACS) analysis.

For immunofluorescent staining, stably transfected cells were cultured for 24 h and fixed in 4% paraformaldehyde in PBS on ice to generate a non-permeabilized condition. For the generation of a permeabilized condition, cells were post-fixed in ice-cold methanol-acetone mixture (1:1). Cells in both conditions were incubated with primary (anti-HA tag and anti-β−actin) followed by fluorescein-conjugated secondary antibodies.

### Generation and Characterization of Transgenic Mice

A full-length cDNA encoding mouse wild-type FGFR3b (Courtesy of Dr. Michael J. Hayman of State University of New York at Stony Brook;^47^) was subjected to site-directed mutagenesis to (i) add a hemagglutinin (HA) tag to facilitate specific detection and (ii) create the S243C mutation (equivalent to human S249C) using a strategy similar to that for the human FGFR3b (see above). After verification by sequencing, the cDNA containing the mouse FGFR3b-S243C was inserted between a 0.6-kb tetracycline response element (TRE) and a 0.6-kb mouse protamine 1 (MP1) gene fragment, the latter of which provided a poly A signal. After the chimeric fragment was confirmed by restriction digestion and sequencing, the 3.7-kb chimeric gene fragment was purified and microinjected into the pronuclei of fertilized eggs from the FVB/N inbred strain. Founders bearing the transgene were identified by genomic PCR with forward primer 5′-GCAGAGCTCGTTTAGTGAACC-3′ and reverse primer: 5′-GTCCTCCCCATCTTCGTCAT-3′. All animal experiments on were carried out according to federal and local regulations and after approval from the Institutional Animal Care and Use Committee of New York University School of Medicine.

The expression of FGFR3b-S243C at the mRNA level was verified by RT-PCR. Briefly, urothelial cells were scrapped off from inside-out bladders and immediately suspended in RNA extraction buffer (Invitrogen). Double-stranded cDNAs were then prepared using standard protocols and used as a template for RT-PCR. The presence of the mutation site was confirmed by direct sequencing of the RT-PCR products.

The expression levels of p16 and p19 were assessed using quantitative real-time PCR with a QuantiTect SYBR-Green PCR kit (Qiagen). Oligonucleotide primer pairs used were (i) for p16: forward, 5′-AGTCCGCTGCAGACAGACTG-3′ and reverse, 5′-CGGGAGAAGGTAGTGGGGTC-3′; (ii) for p19: forward, 5′-CTTGGTCACTGTGAGGATTC-3′ and reverse, 5′-CACGTACTCTCCTCCCCTCA-3′. PCR conditions were 95 °C for 3 min for the first cycle; 94 °C for 30 s, 55–58 °C for 30 s and 72 °C for 1.5 min for 50 cycles. Amplification of mouse β-actin was carried out in parallel for each sample and used as an internal reference.

### Histopathology, immunohistochemistry and Western blotting

Freshly dissected urinary bladders were fixed in 10% PBS-buffered formalin and processed routinely for hematoxylin and eosin staining prior to microscopic examination. For antibody staining, deparaffinized sections were microwaved at maximal power in a citrate buffer (pH 6.0) for 20 min. The primary antibodies used in this study were anti-HA (Abcam, Inc., 1:1,000), anti-β-actin (Sigma, 1:2,000), anti-E-cadherin (SCBT Inc., 1:400), anti-p-MAPK (Thr202/Tyr204; Cell Signaling Technology, Inc., 1:2,000) anti-p-AKT (Ser473; Cell Signaling Technology, Inc., 1:200), anti-p-S6 (Ser235/Ser236; Cell Signaling Technology, Inc., 1:400) and anti-p-STAT (Tyr705; Cell Signaling Technology, Inc., 1:400). Western blotting was carried out using total urothelial proteins prepared in 20 mM Tris-HCl (pH 7.5) containing 10% SDS, 50 mM NaCl, 5 mM β-mercapto-ethanol and a cocktail of protease inhibitors. After SDS-PAGE, proteins were electrophoretically transferred onto PVDF membrane and incubated with primary and then HRP-conjugated secondary antibodies. The membrane was developed by enhanced chemiluminescence (Amersham Biosciences). The primary antibodies used (all from Cell Signaling Technology, Inc., unless otherwise noted or differ from the immunohistochemistry) were: anti-HA (1:1,000), anti-p-MAPK (1:3,000), anti-MAPK (1:2,000), anti-p-AKT (S473; 1:1,000), anti-AKT (1:1,000), anti-p16 (Abcam, Inc., 1:1,000), anti-p19 (EMD Millipore, 1:500), anti-p53 (SCBT Inc., 1:500) and anti-p21 (Abcam, Inc., 1:500). Antibody against β-actin (Sigma, 1:5,000) served as a loading control for all experiments.

### Statistical Analysis

Student *t* test (two-tailed) was employed for evaluating the statistical significances in (i) the growth-promotion capacities in UMUC3 cells between FGFR3b-S249C and wild-type FGFR3b; (ii) the expression levels of p16 and p19 between doxycycline-treated mice expressing FGFR3b-S249C and untreated mice not expressing FGFR3b-S249C; and (iii) the frequency of high-grade, papillary urothelial carcinoma between doxycycline-treated and untreated triple transgenic mice.

## Additional Information

**How to cite this article**: Zhou, H. *et al*. FGFR3b Extracellular Loop Mutation Lacks Tumorigenicity *In Vivo* but Collaborates with p53/pRB Deficiency to Induce High-grade Papillary Urothelial Carcinoma. *Sci. Rep*. **6**, 25596; doi: 10.1038/srep25596 (2016).
